# Supplementary material for: Computed and Subjective Blue Scleral Color Analysis as a Diagnostic Tool for Iron Deficiency: A Pilot Study
Source: J Clin Med. 2019 Nov 5;8(11):1876. doi: 10.3390/jcm8111876 (PMC6912357; doi:10.3390/jcm8111876)
Supplement: Supplementary file 1 [file jcm-08-01876-s001.pdf]

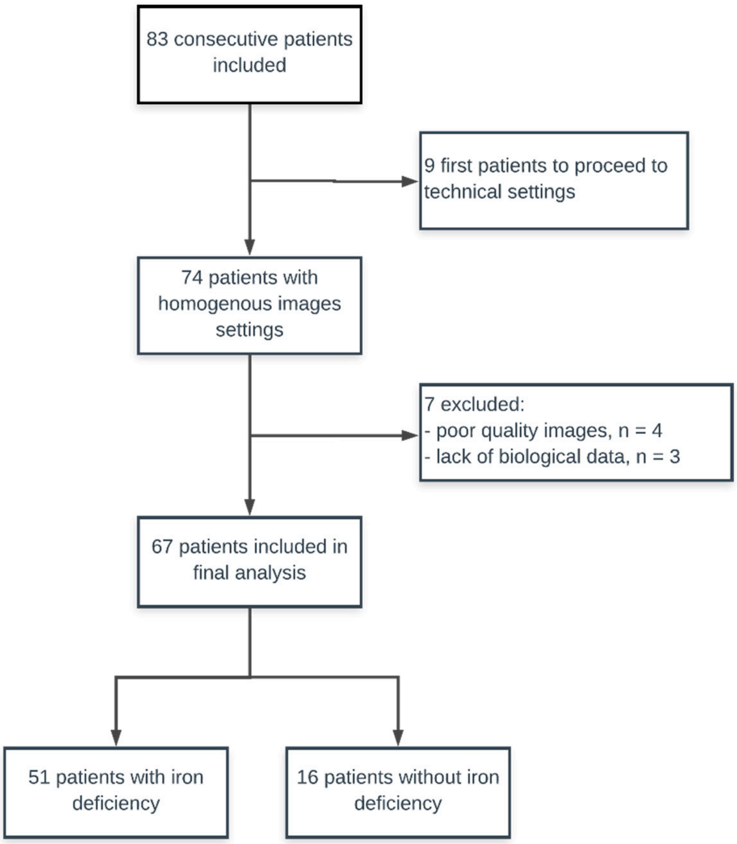

Figure S1. Flowchart.

Table S2. Baseline characteristics of population.

	**All (n = 67)**	**ID (n = 51)**	**NID (n = 16)**
Male (%)	30 (44%)	19 (37%)	11 (68%)
Mean age (yr)	59.9 ± 20.1	63.1 ± 20.3	49.6 ± 16.4
BMI (kg/m²)	26.2 ± 7.3	26.7 ± 7.8	24.5 ± 5.6
Dark iris, n (%)	43 (64%)	35 (68%)	8 (50%)
Hemoglobin (g/dL)	10.3 ± 2.5	9.7 ± 2.1	12 ± 3
No. of patients with anemia (male)	53 (23)	46 (19)	7 (4)
Mean corpuscular volume (fL)	84.4 ± 8.5	82.4 ± 7.8	90.5 ± 8
Platelet count (G/L)	255 ± 116	267 ± 113	217 ± 119
Ferritin (µg/L)	28.5 (16;75)	20.5 (11;34)	244.5 (111.7;440.7)
TPS (%)	9.6 (6;38.5)	8 (6;11)	40 (32.5;54)
Fibrinogen (g/L)	4.2 ± 1.8	4.2 ± 1.6	4.2 ± 2.8
C-reactive protein (g/L)	9.6 (2.9;37.5)	13.5 (3.5;43)	2.9 (2.9;9)

Data are presented as mean ± standard deviation. Ferritin, TPS and C-reactive protein are presented as median and Inter-Quartile-Range. TPS: Transferrin Percent Saturation.
